# Draft Genome Sequence of *Donghicola* sp. Strain KarMa, a Model Organism for Monomethylamine-Degrading Nonmethylotrophic Bacteria

**DOI:** 10.1128/genomeA.01623-16

**Published:** 2017-02-16

**Authors:** Karsten Zecher, Marcel Suleiman, Daniel Wibberg, Anika Winkler, Bodo Philipp, Jörn Kalinowski

**Affiliations:** a Institute of Molecular Microbiology and Biotechnology, University of Münster, Münster, Germany; b Center for Biotechnology (CeBiTec), Bielefeld University, Bielefeld, Germany

## Abstract

The C_1_-compound monomethylamine can serve as a nitrogen, carbon, and energy source for heterotrophic bacteria. The marine alphaproteobacterium *Donghicola* sp. strain KarMa can use monomethylamine as a source only for nitrogen and not for carbon. Its draft genome sequence is presented here and reveals putative gene clusters for the methylamine dehydrogenase and the *N*-methylglutamate pathways for monomethylamine metabolism.

## GENOME ANNOUNCEMENT

Methylamines (e.g., monomethylamine [MMA]) are ubiquitous nitrogen compounds within marine habitats and are derived from the degradation of proteins and other organic nitrogen compounds (1, 2). As the simplest primary amine, MMA can be degraded by Gram-negative bacteria via two main pathways (3). MMA can be degraded by oxidative deamination to formaldehyde and ammonium via a periplasmic pyrroloquinoline-dependent dehydrogenase protein complex encoded by the *mau* gene cluster (4). Alternatively, several Gram-negative bacteria are known to bind MMA to glutamate under the release of ammonium yielding the characteristic intermediate *N*-methylglutamate (NMG) (5). NMG is than cleaved by NMG dehydrogenase to formaldehyde and glutamate. The MMA dehydrogenase pathway is commonly found in methylotrophic bacteria. Nonmethylotrophic bacteria, which use ammonium only and cannot assimilate formaldehyde, do frequently use the NMG pathway.

*Donghicola* sp. strain KarMa was enriched by cocultivation with the diatom *Phaeodactylum tricornutum* strain UTEX 646 with MMA as the sole nitrogen, carbon, and energy source for the bacterium; physiological analysis revealed that strain KarMa can use MMA as a nitrogen source only, whereas it cannot assimilate the carbon (6). Genomic DNA was extracted from MMA and glucose-grown cells of strain KarMa with a blood and cell culture DNA minikit (Qiagen).

Genomic DNA was sequenced on the MiSeq system (Illumina). A paired-end sequencing run in combination with an 8-kb mate-pair sequencing run yielded 2,030,551 sequence reads accounting for 400,825,012 bp of total sequence information. Thus, a 92-fold coverage was achieved for the approximately 3.3-Mb *Donghicola* sp. strain KarMa genome. After assembly of all sequence reads by applying the GS *De Novo* Assembler version 2.8 software, the draft genome consisted of eight scaffolds, representing five replicons. For assembly validation, an *in silico* strategy was applied (7). Sizes (and G+C content) of the draft chromosome and the draft plasmids pDKa, pDKb, pDKc, and pDKd are 3,262,507 bp (59.37%), 569,630 bp (57.58%), 278,491 bp (57.86%), 96,696 bp (60.07%), and 6,286 bp (59.58%), respectively.

The annotation of the genome was done within the GenDB platform (8). The draft genome of the strain harbors 4,255 protein-coding sequences, as well as 45 tRNA genes and five rRNA operons. In addition, the genome of strain KarMa harbors all putative genes necessary for degradation of MMA by the *mau* as well as the NMG pathway. Further, several putative genes possibly involved in interaction with diatoms (dimethylsulfoniopropionate, glycolate, polysaccharide, and amino acid catabolism) were identified.

### Accession number(s).

This whole-genome shotgun project has been deposited in the EMBL/GenBank database (EBI, NCBI) under the accession numbers FMJB01000001 to FMJB01000066.
